# Carbon monoxide poisoning is associated with increased risk of migraine in the long term: a nationwide population-based cohort study

**DOI:** 10.3389/ftox.2025.1532584

**Published:** 2025-01-22

**Authors:** Heewon Hwang, Solam Lee, Yeon-Woo Heo, Woo-Seok Ha, Kyung Min Kim, Yong Sung Cha

**Affiliations:** ^1^ Department of Neurology, Yonsei University Wonju College of Medicine, Wonju, Republic of Korea; ^2^ Department of Dermatology, Yonsei University Wonju College of Medicine, Wonju, Republic of Korea; ^3^ Department of Neurology, Epilepsy Research Institute, Yonsei University College of Medicine, Seoul, Republic of Korea; ^4^ Department of Emergency Medicine, Yonsei University Wonju College of Medicine, Wonju, Republic of Korea; ^5^ Research Institute of Hyperbaric Medicine and Science, Yonsei University Wonju College of Medicine, Wonju, Republic of Korea

**Keywords:** headache, migraine, population-based cohort study, carbon monoxide poisoning, hyperbaric oxygen therapy

## Abstract

**Objective:**

Carbon monoxide poisoning can cause migraine-like attacks. However, the association between carbon monoxide poisoning and the risk of migraine has not been thoroughly studied. This study aimed to investigate the long-term risk of migraine in patients with carbon monoxide poisoning.

**Methods:**

This nationwide, population-based cohort study was conducted using the administrative database of the National Health Insurance Service of Korea from 2002 to 2021. Patients with carbon monoxide poisoning with at least one visit documented according to the International Classification of Diseases, 10th Revision code T58 were included. Patients were only included if they had the same diagnostic code at two or more outpatient clinic visits. The primary outcome of this study was the incidence of migraine after carbon monoxide poisoning.

**Results:**

The overall risk of migraine was higher in the carbon monoxide poisoning group regardless of age, sex, or use of hyperbaric oxygen therapy (adjusted hazard ratio, 1.37; 95% confidence interval, 1.28–1.48). The carbon monoxide poisoning group had a persistently higher cumulative incidence of migraine during the observation period than the control group.

**Conclusion:**

Carbon monoxide poisoning was associated with an increased overall risk of developing migraine during long-term follow-up.

## 1 Introduction

In the United States, approximately 50,000 patients are admitted to hospital emergency departments annually due to carbon monoxide (CO) poisoning, resulting in 1,500 deaths (Weaver and Moon, 2019; Hampson, 2016). Recent data from South Korea reported a prevalence rate of 8.64 CO poisoning cases per 10,000 people, with a steady increase from 2010 to 2019, during which the availability of hyperbaric oxygen therapy (HBO2) for CO poisoning patients doubled nationwide (Han et al., 2023). CO poisoning can lead to various neurological effects, including headaches (Ning et al., 2020; Pages et al., 2014; Meng et al., 2023; Hampson, 2002).

Migraine, affecting around one billion people globally, is the second most common neurological disorder after tension-type headache (Safiri et al., 2022). In the Korean population, the 1 year prevalence rates of migraine were 6.0% and 5.2% in the 2009 and 2018 surveys, respectively (Kim et al., 2021). Diagnosis is based on the clinical criteria outlined in the International Classification of Headache Disorders, third edition (ICHD-3), including recurrent headache attacks of moderate-to-severe pain lasting 4–72 h, often accompanied by nausea, vomiting, photophobia, and phonophobia (The International Classification of Headache Disorders, 2013).

According to ICHD-3, a CO-induced headache is typically a bilateral headache of varying intensity that arises within 12 h of CO exposure and resolves within 72 h. In cases of acute CO poisoning, these headaches can escalate to severe intensity, causing symptoms like nausea and vomiting that disrupt daily activities (The International Classification of Headache Disorders, 2013). While the primary mechanism of CO-induced headache was previously attributed to reduced oxygen-carrying capacity leading to hypoxia, recent research by Arngrim et al. (2014) suggest that CO exposure may also affect migraine pathways through nitric oxide signaling and activation of cyclic guanosine monophosphate.

The International Headache Society notes that CO poisoning can trigger headaches, with symptom severity correlating with carboxyhemoglobin (COHb) levels: mild headaches at COHb levels of around 10%, moderate pulsating headaches at 20%–30%, and severe headaches with nausea, vomiting, and blurred vision at 30%–40% (The International Classification of Headache Disorders, 2013). In a prior study involving 100 CO-poisoned patients with acute headaches, where the mean COHb level was 21.3%, 66% of patients reported frontal pain, and 41% described their headaches as throbbing (Hampson, 2002).

Although research has explored acute headaches after CO exposure, there remains a gap in understanding the long-term headache outcomes following CO poisoning (Ghanizada et al., 2018). Thus, this study aims to examine the potential association between CO poisoning and the subsequent development of migraine in patients without a prior history of headaches.

## 2 Materials and methods

### 2.1 Data source

This nationwide population-based cohort study utilized the administrative database of the National Health Insurance Service (NHIS) of Korea from 1 January 2002 to 31 December 2021, or until the time of an individual’s death (Cheol et al., 2017). The NHIS covers more than 99% of the country’s population. This extensive database contains details encompassing demographics, socioeconomic backgrounds, diagnoses recorded according to the ICD-10, as well as medical treatment records of a vast cohort exceeding 50 million individuals.

### 2.2 Ethics statement

This study was conducted in accordance with the Strengthening the Reporting of Observational Studies in Epidemiology Reporting Guidelines. (von Elm et al., 2007). This study was approved by the Korean National Institute for Bioethics Policy (NHIS-2022-1-366) and Wonju Severance Christian Hospital’s Institutional Review Board (approval number: CR321347), and adhered strictly to the principles outlined in the Declaration of Helsinki. Since this study used de-identified data from the NHIS, the ethics committee of our Institutional Review Board waived the requirement for informed consent from patients.

### 2.3 Study population

**FIGURE 1:**
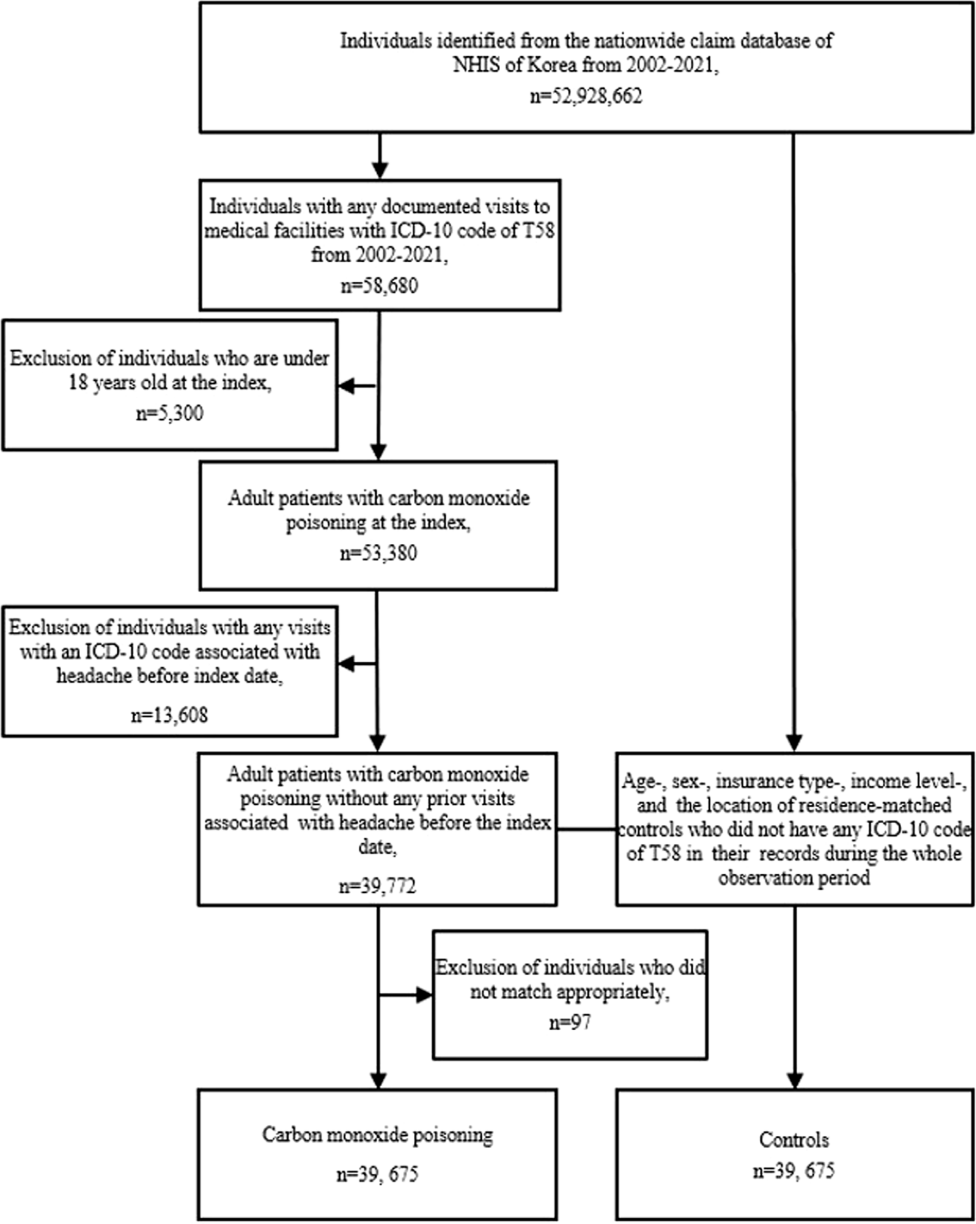
Flowchart of study population selection. Abbreviations: ICD-10 International Classification of Disease, 10th revision, NHIS National Health Insurance Service.

This study analyzed data from the NHIS database from 2002 to 2021. The study categorized the individuals into two groups: a CO poisoning group and a control group. The CO poisoning group consisted of individuals aged 18 years or older who had recorded visits to medical facilities with an ICD-10 code of T58 as either the principal diagnosis or an adjuvant diagnosis between 1 January 2002 and 31 December 2021. The control group comprised individuals who were matched in a 1:1 ratio in terms of age, sex, insurance type, income level, and residence location but had no record of ICD-10 code T58 in their medical history during the observation period (Figure 1).

For both groups, the index date was defined as the date of the documented visit for CO poisoning in patients and was identical for their matched controls. Subsequent follow-ups began on the index date and continued until any of the following events occurred: death, migration, or conclusion of the observation period (31 December 2021), whichever occurred first.

### 2.4 Validating the criteria used to identify patients with CO poisoning

We initially evaluated the reliability of the criteria used to identify patients with CO poisoning using an administrative database. We applied these identical criteria to the electronic medical records of patients who visited our facility for medical care on CO poisoning from January 2006 to August 2022. Subsequently, two board-certified emergency medicine specialists thoroughly confirmed the diagnosis, and the positive predictive value was determined based on these criteria.

### 2.5 Variables of baseline characteristics

The study collected data from database records, encompassing information regarding age at the index date, sex, type of health insurance, income level, residence region, and pre-existing comorbidities. Pre-existing comorbidities, including hypertension, diabetes mellitus, dyslipidemia, liver disease, chronic kidney disease, stroke, central nervous system (CNS) tumors, CNS infection, dementia, and Parkinson’s disease, were identified based on their respective ICD-10 codes.

The Charlson Comorbidity Index (CCI) score was calculated on the index date. The CCI score is one of the indices used to predict outcomes such as mortality by measuring the severity of various comorbid medical conditions in a patient. This is particularly useful when adjusting for multiple confounding variables in observational studies (Bannay et al., 2016). A higher CCI score indicates a greater burden of comorbidities. CCI score comprises comorbid conditions, including myocardial infarction, congestive heart failure, peripheral vascular disease, cerebrovascular disease, dementia, chronic pulmonary disease, connective tissue disease, peptic ulcer disease, liver disease, diabetes without complications, diabetes with end-organ damage, hemiplegia or paraplegia, renal disease (mild to moderate), any tumor including leukemia and lymphoma except malignant neoplasm of the skin, moderate to severe liver disease, metastatic solid tumor (cancer), and human immunodeficiency virus-acquired immunodeficiency (Charlson et al., 1987).

**FIGURE 2:**
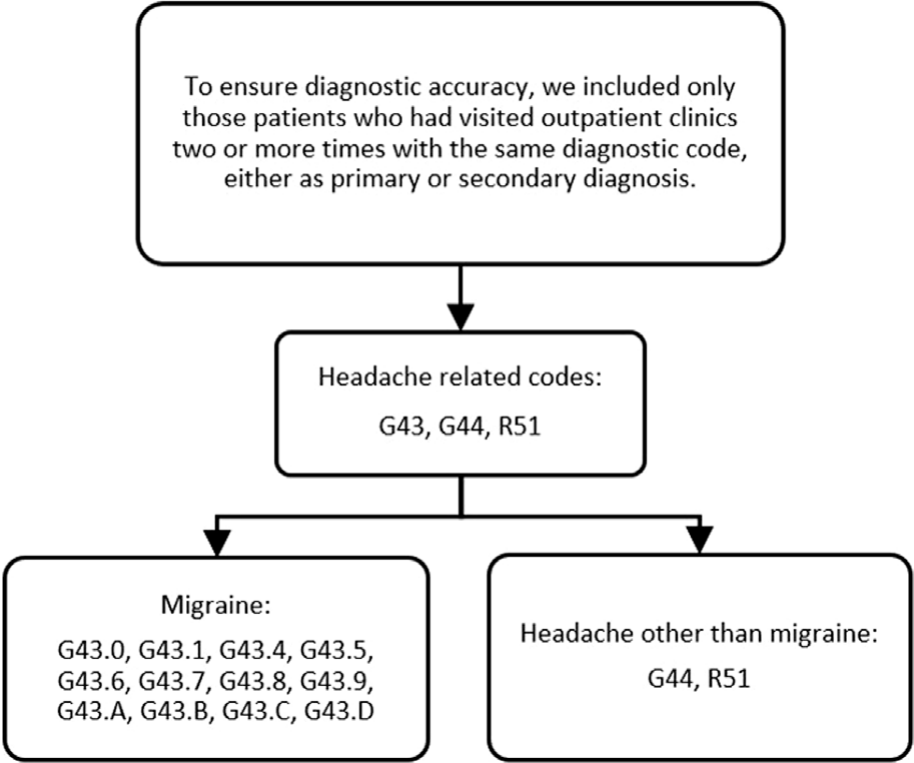
Operational diagnosis of headache-related codes, migraine, and headache other than migraine. Note: G43.0, migraine without aura; G43.1, migraine with aura; G43.4, hemiplegic migraine; G43.5, persistent migraine aura without cerebral infarction; G43.6, persistent migraine aura with cerebral infarction; G43.7, chronic migraine without aura; G43.8, other migraine; G43.9, migraine, unspecified; G43. A, cyclical vomiting; G43. B, ophthalmoplegic migraine; G43. C, periodic headache syndromes in child or adult; G43. D, abdominal migraine; G44, other headache syndromes; R51, headache.

Migraine was identified based on the following ICD-10 codes: G43.0, G43.1, G43.4, G43.5, G43.6, G43.7, G43.8, G43.9, G43. A, G43. B, G43. C, and G43.D. Headaches other than migraine were identified based on the ICD-10 codes G44 and R51 (Figure 2).

### 2.6 Study outcome measurement

The primary outcome of this study was the incidence of migraine in patients with CO poisoning and controls. To ensure diagnostic accuracy, we included only patients who visited outpatient clinics two or more times with the same diagnostic code, either as a primary or a secondary diagnosis. Patients diagnosed using headache-related codes (G43, G44, R51) before the index date were excluded. The control group was also sampled from a population that was not diagnosed with any head-related codes. We conducted control-group sampling after finalizing the selection of test subjects to appropriately match test subjects and controls (Figure 1).

The secondary outcome was the relative risk of migraine in patients with CO poisoning, depending on the presence or absence of HBO2. The HBO2 group was confirmed through the presence of NHIS procedural codes M0581–M0588 and M5861–M5868 at the time of CO poisoning. Patients without a diagnosis of CO poisoning on the index date were not included.

### 2.7 Statistical analysis

The baseline demographic characteristics of the study population are described using frequencies with percentages or means with standard deviations. To assess the differences between the two groups, independent t-tests and chi-squared tests were used to analyze the clinical characteristics.

The incidence rates of the various outcomes were calculated as the number of occurrences per 10,000 person-years. Hazard ratios and 95% confidence intervals comparing the CO poisoning and control groups throughout the study period were determined using multivariate Cox proportional hazards regression analyses.

In the multivariate Cox proportional hazards regression analyses, the following covariates were considered: birth year, sex, index date, insurance type, income level, residential location, CCI score at index date, CNS infection, and Parkinson’s disease. Diagnoses of CNS infection (coded as G04 or G05) and Parkinson’s disease (coded as G20) were determined based on ICD-10 codes. Cases of other neurological conditions, including cerebrovascular disease and dementia, were already included as components of the CCI score; therefore, we did not perform duplicate adjustments.

These analytical procedures were repeated for subgroup analyses, considering the following strata: 1) sex (male/female); 2) age (<40 years/40–59 years/≥60 years); 3) history of stroke, neurodegenerative diseases, and CNS tumor or infection at the index date; and 4) treatment history with HBO2 for CO poisoning.

All statistical analyses were performed using SAS (version 9.4; SAS Institute, Cary, NC) and R (version 3.6.3; R Foundation, Vienna, Austria) at a significance level of 5%.

## 3 Results

### 3.1 Validating the criteria used to identify patients with CO poisoning

According to our defined criteria, we identified 1,991 individuals who visited our institution between January 2006 and August 2022 and received at least one ICD-10 code for T58. Among them, 1,920 patients were confirmed to have CO poisoning in our review. The positive predictive value was 96.4% (95% confidence interval, 95.5%–97.2%), demonstrating the accuracy with which our criteria were able to identify patients with CO poisoning in the NHIS database.

### 3.2 Characteristics of the study population

For the CO poisoning group, the mean follow-up period was 5.9 ± 4.4 years, whereas the control group had a mean follow-up period of 6.6 ± 4.5 years. This study included 39,675 patients with CO poisoning, and 39,675 age-, sex-, insurance type-, income level-, and residential location-matched controls. Both the patient and control groups were 38.9% female, and the mean age at the index date was 44.2 ± 16.7 years old for both groups. The lowest income level was the most prevalent, accounting for 31.6% of the distribution, followed by the highest income level at 28.9%. Residences were rural for 59.0% of participants and urban for 41.0%, demonstrating a well-matched geographic distribution.

Analysis of comorbidities revealed differences between the two groups. Patients with CO poisoning exhibited a higher prevalence of various comorbid conditions at the index date compared to controls, including hypertension (20.1% vs 18.9%, P < 0.001), diabetes mellitus (12.4% vs 10.8%, P < 0.001), dyslipidemia (26.1% vs 23.5%, P < 0.001), liver disease (26.6% vs 21.7%, P < 0.001), stroke (3.3% vs 2.1%, P < 0.001), CNS tumor (0.1% vs 0.03%, P < 0.001), CNS infection (0.1% vs 0.02%, P < 0.001), dementia (2.1% vs 1.5%, P < 0.001), and Parkinson’s disease (0.4% vs 0.2%, P < 0.001).

A lower proportion of patients in the CO poisoning group had a CCI score of 0 than in the control group (46.9% vs 51.4%, P < 0.001), indicating a higher burden of comorbidities in the former at the index date. In the CO poisoning group, 20.2% of patients received HBO2, whereas no patients in the control group underwent this treatment. The incidence of death in the CO poisoning group was 13.2%, indicating an unfavorable prognosis compared with the control group, which had a mortality rate of 2.2% (Table 1).

**TABLE 1:** Baseline characteristics of the study population.

	Value (count [%] or mean ± SD)		*P*-value
Characteristic	Carbon monoxide poisoning	Controls	
Number of subjects	39,675 (100%)	39,675 (100%)	
Female sex, n (%)	15,427 (38.9%)	15,427 (38.9%)	
Age at index, years	44.2 ± 16.7	44.2 ± 16.7	
Comorbidities, n (%)			
Hypertension	7,992 (20.1%)	7,506 (18.9%)	<0.001
Diabetes mellitus	4,925 (12.4%)	4,290 (10.8%)	
Dyslipidemia	10,341 (26.1%)	9,328 (23.5%)	
Liver disease	10,553 (26.6%)	8,589 (21.7%)	
Chronic kidney disease	266 (0.7%)	257 (0.7%)	
Stroke	1,292 (3.3%)	850 (2.1%)	
CNS tumor	18 (0.1%)	11 (0.03%)	
CNS infection	21 (0.1%)	6 (0.02%)	
Dementia	846 (2.1%)	576 (1.5%)	
Parkinson disease	174 (0.4%)	97 (0.2%)	
CCI score at index date	1.5 ± 2.0	1.4 ± 1.9	
CCI score 0	18,599 (46.9%)	20,406 (51.4%)	
Hyperbaric oxygen, n (%)			
No	31,649 (79.8%)	39,675 (100%)	
Yes	8,026 (20.2%)	0	
Mean follow up period (years)	5.9 ± 4.4	6.6 ± 4.5	
Death, n (%)	5,218 (13.2%)	865 (2.2%)	<0.001

Abbreviations: SD, standard deviation; CNS, central nervous system; CCI, charlson comorbidity index.

### 3.3 Risk of migraine

**FIGURE 3:**
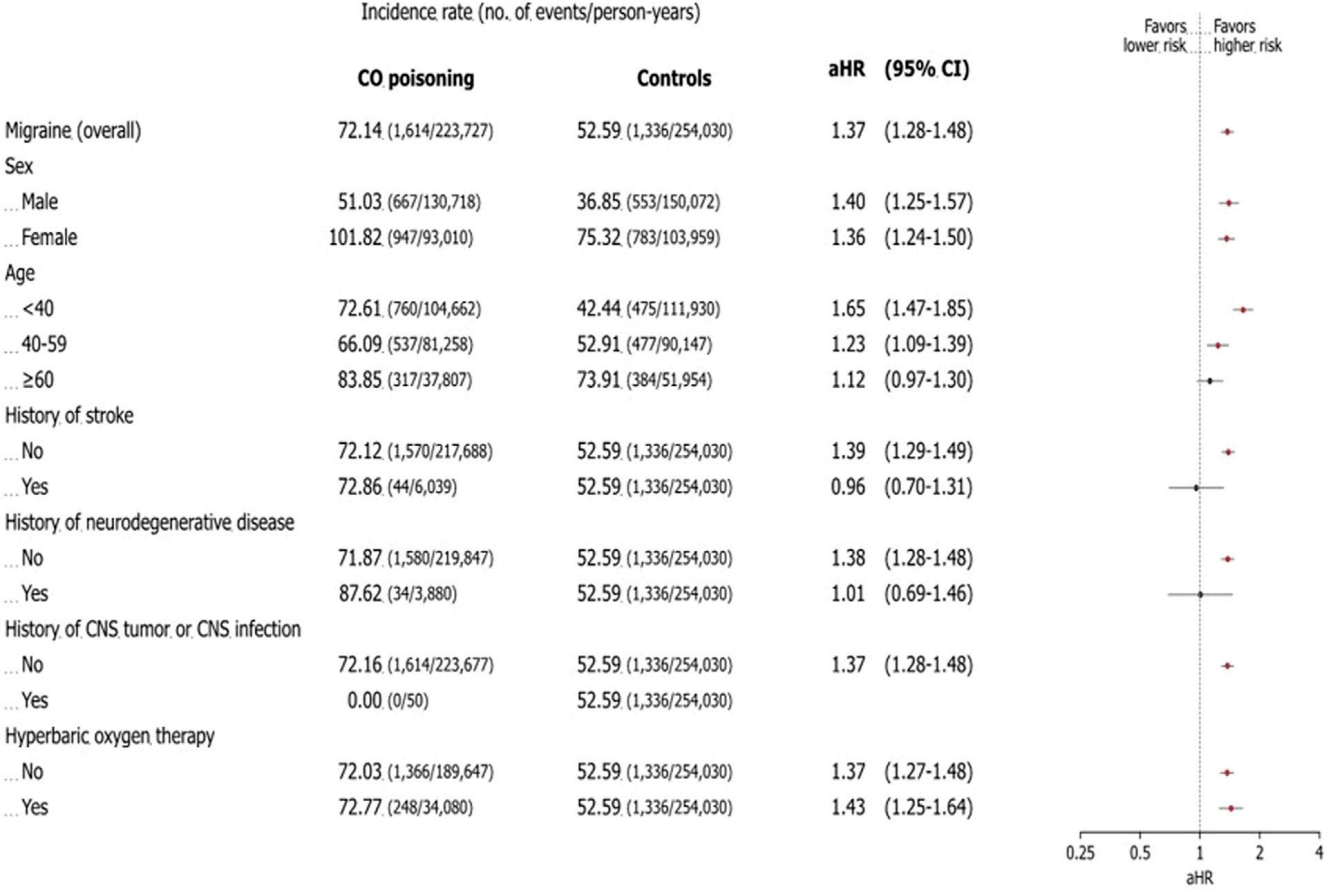
Association between risk of migraine and carbon monoxide poisoning: subgroup analysis. The forest plot shows the adjusted hazard ratio and 95% confidence interval for each outcome in patients with carbon monoxide poisoning and controls during the study period. The incidence rate is the number of deaths divided by 100,000 person-years. The plot presents the statistical estimates from the multivariable Cox proportional hazards regression analysis in which birth year, sex, insurance type, income level, residence location, Charlson comorbidity index score, central nervous system infection, and Parkinson’s disease at the index date are adjusted. The subgroup analyses are performed using the selected variables. Abbreviations: CO carbon monoxide, aHR adjusted hazard ratio, CNS central nervous system.

**FIGURE 4:**
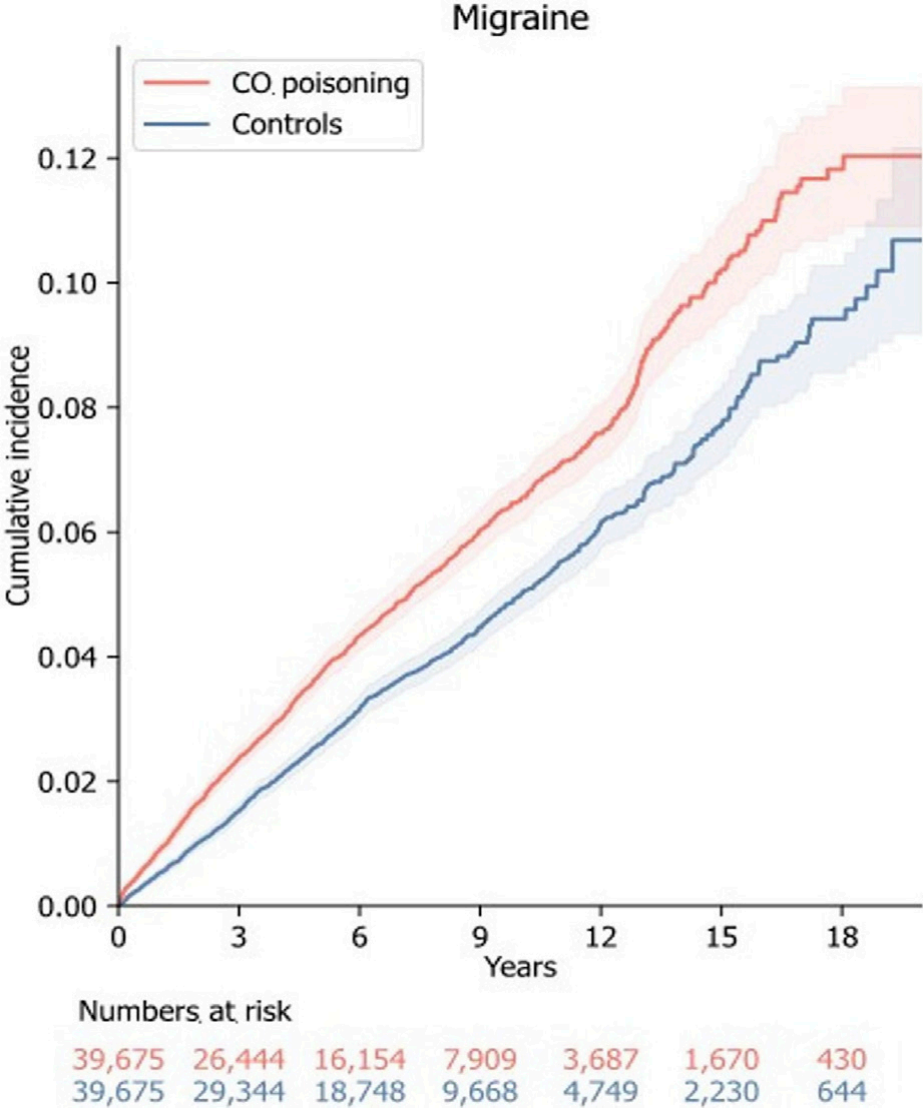
The cumulative incidence plot for migraine. The cumulative incidence plot shows the cumulative incidence functions and number of events in patients with carbon monoxide poisoning and controls. The shaded area shows the 95% confidence interval of the cumulative incidence function. Abbreviations: CO carbon monoxide.

After adjusting for factors such as birth year, sex, index date, insurance type, income level, residential location, CCI score at the index date, history of CNS infection, and Parkinson’s disease, the CO poisoning group had a significantly higher risk of migraine, with an adjusted hazard ratio of 1.37 (95% confidence interval: 1.28–1.48) (Figure 3). Throughout the observation period, the cumulative incidence of migraine remained consistently higher in the CO poisoning group compared to the control group, with the difference becoming even more pronounced around 12 years after the index date (Figure 4).

### 3.4 Subgroup analyses

Subgroup analyses by sex, age, history of stroke, neurodegenerative disease, CNS tumor or infection, and HBO2 use showed no significant difference in migraine risk based on sex. However, both males and females had an elevated risk of migraine (males: adjusted hazard ratio 1.36; 95% confidence interval: 1.25–1.57; females: adjusted hazard ratio 1.36; 95% confidence interval: 1.24–1.50).

Among age groups, patients exposed to CO poisoning before age 40 had the highest risk of developing migraine, with an adjusted hazard ratio of 1.65 (95% confidence interval: 1.47–1.85). In comparisons between groups with and without a history of stroke and neurodegenerative disease, those without such a history showed a higher risk of migraine. The adjusted hazard ratio for migraine was 1.39 (95% confidence interval: 1.29–1.49) in the no-stroke group compared to 0.96 (95% confidence interval: 0.70–1.31) in the stroke group, and 1.38 (95% confidence interval: 1.28–1.48) in the no-neurodegenerative disease group compared to 1.01 (95% confidence interval: 0.69–1.46) in the neurodegenerative disease group. The use of HBO2 therapy did not significantly affect the long-term risk of migraine, with an adjusted hazard ratio of 1.37 (95% confidence interval: 1.27–1.48) in treated patients compared to 1.43 (95% confidence interval: 1.25–1.64) in untreated patients (Figure 3).

## 4 Discussions

In this nationwide population-based cohort study, the overall risk of migraine was 1.37-fold higher in the CO poisoning group compared to the control group. The increased risk of migraine in the CO poisoning group was consistent with subgroup analyses. An increment in cumulative incidence was seen from the index date, and noticeable divergence became evident after 12 years from the index date according to the incidence plot of this study. This finding suggests that CO poisoning may potentially contribute to intrinsic differences that continue to trigger migraine or migraine-like headaches in the long term after an event in individuals with no history of headaches.

While previous research has examined the occurrence of headaches during the acute phase of CO poisoning, few studies have investigated its long-term effects as a possible trigger for migraine. Studies indicate that headaches can occur relatively soon after CO exposure. (Hampson, 2002). Studies have suggested that headaches occurring during the winter season may be associated with acute, accidental CO exposure from sources such as heaters and kitchen stoves (Heckerling, 1987; Keleş et al., 2008). Recent studies have indicated that in many regions of South Korea, coal briquette boilers, which have been extensively used for heating over a long period, significantly contribute to CO poisoning prevalence, while the rise in outdoor activities like camping is also emerging as a new risk factor (Han et al., 2023). In a randomized, double-blind, placebo-controlled study conducted by Ghanizada et al., found that inhaling CO to a 22% COHb level could trigger peak headaches hours later (Ghanizada et al., 2018). Additionally, a Turkish case report suggested that wintertime migraine attacks may be symptomatic of CO poisoning (Kanburoglu et al., 2014). In 1995, Ely and Moorehead (1995) reported 30 patients with chronic headaches up to 24 months following CO intoxication, highlighting the potential for persistent symptoms.

CO has a notably high binding affinity to heme over 200 times greater than oxygen which reduces the blood’s oxygen-carrying capacity and leads to hypoxia in oxygen-demanding tissues, particularly in the heart and brain. Previous studies have shown that experimentally induced hypoxia can trigger migraine-like headaches in individuals who experience migraine with aura (Arngrim et al., 2016). Based on these findings, we hypothesized that hypoxia from CO poisoning could serve as a mechanism for triggering migraines. However, no reliable biomarker currently exists to indicate hypoxia specifically from CO poisoning, and given the retrospective nature of our study, establishing causality was not feasible.

CO has also recently been identified as a pain-modulating neurotransmitter (Verma et al., 1993). Endogenously produced, it influences pain transmission through the cyclic guanosine monophosphate pathway, exhibiting a strong affinity for heme within soluble guanylate cyclase. This affinity activates soluble guanylate cyclase, increasing cyclic guanosine monophosphate production and glutamate release (Morita et al., 1995). In addition, CO interacts with nitric oxide, which plays a crucial role in the mechanisms underlying migraine generation. (Mustafa et al., 2009). This interaction results in the dilation of cerebral vessels and the generation of free radicals, causing headaches (Barkoudah et al., 2004; Leffler et al., 2001). Further basic research is needed on the process and mechanism by which CO triggers migraines.

While HBO2 therapy can alleviate acute-phase headaches from CO poisoning (Ouahmane et al., 2023), it did not appear to impact the long-term development of migraines in our study. However, limitations in retrospective national cohort data, such as lack of detailed information on the duration and intensity of HBO2 treatment, may have affected these findings.

There is still a lack of research on whether CO poisoning can result in migraines as a long-term complication. This study highlights the need for future research on migraines as a potential long-term outcome of CO poisoning, although it has limitations. First, as a retrospective observational study, it cannot establish causal relationships. Future research is needed to investigate the pathophysiological effects of CO on migraine development. Second, using diagnostic codes from a national administrative database might have led to migraine misclassification. Without detailed chart reviews, including personal or family history of migraine, it is challenging to confirm whether these were true migraines or migraine-like headaches. (Kanburoglu et al., 2014; Lipton et al., 1997). Third, confounding factors of chronic CO exposure, such as smoking history, long-term air pollution exposure, and CO poisoning severity at the index date, were not examined, warranting further research. Although we attempted to account for differences in comorbidity profiles using the CCI score, residual confounding factors may still have influenced the development of migraines. Additionally, potential bias may have been introduced by excluding subjects with missing data, and the impact of baseline comorbidities cannot be fully disentangled solely through statistical adjustments. Fourth, the study was conducted on a single ethnicity, emphasizing the need for further validation to assess the external generalizability. Lastly, we did not distinguish between migraine with and without aura. Since this study was conducted using national health insurance data, a concise profile of headaches could not be obtained. Therefore, acknowledging the limitations of these data and to avoid overinterpretation, we enrolled patients with migraines in general without distinguishing between those with and without aura. This generalization limits our ability to draw detailed conclusions about the long-term effects of CO poisoning on each type and suggests that further research is needed to clarify any differences in impact.

## 5 Conclusion

In this nationwide cohort study, a noteworthy association was observed between CO poisoning and an elevated overall risk of migraine during the long-term monitoring period. Notably, the risk of experiencing migraine was significantly higher in the CO poisoning group than in the control group, regardless of sex, age, or the application of HBO2. These findings suggest that individuals who survive acute CO poisoning should receive long-term management, including ongoing monitoring for the potential onset of migraine.

## Data Availability

The raw data supporting the conclusions of this article will be made available by the authors, without undue reservation.
